# Biochemical profiling provides a low-cost and globally accessible method to detect falsified vaccines and insulin

**DOI:** 10.1038/s41598-026-37281-9

**Published:** 2026-02-16

**Authors:** Jennifer Brook, Tehmina Bharucha, Benediktus Yohan Arman, Céline Caillet, Susan Morris, Michelle Taylor-Siddons, Laura Gomez Fernandez, John Walsby-Tickle, Isabelle Legge, Sneha Banerjee, Michael Deats, Rajender Jena, Dnyanesh S. Ranade, Shrikrishna R. Chunekar, Kundan D. Patil, Sunil Gairola, Susanna Dunachie, Hamid A. Merchant, Robert Stokes, Rutendo Kuwana, Alexandrine Maes, Sarah Gilbert, James McCullagh, Pavel Matousek, Nicole Zitzmann, Paul N. Newton, Bevin Gangadharan, Tim James

**Affiliations:** 1 Department of Clinical Biochemistry, John Radcliffe Hospital, Oxford University Hospitals NHS Foundation Trust, Oxford, OX3 9DU UK; 2 Department of Biochemistry, University of Oxford, Oxford, OX1 3QU UK; 3 Kavli Institute for Nanoscience Discovery, University of Oxford, Oxford, OX1 3QU UK; 4 Medicine Quality Research Group, NDM Centre for Global Health Research, Nuffield Department of Medicine, University of Oxford, Oxford, OX3 7LG UK; 5 Mahidol-Oxford Tropical Medicine Research Unit, Faculty of Tropical Medicine, Mahidol University, Bangkok, 10400 Thailand; 6 Infectious Diseases Data Observatory, Centre of Tropical Medicine & Global Health, Nuffield Department of Medicine, University of Oxford, Oxford, OX3 7LG UK; 7 Pandemic Sciences Institute, Nuffield Department of Medicine, University of Oxford, Oxford, OX3 7TY UK; 8 Hospital Pharmacy, Oxford University Hospitals NHS Foundation Trust, Oxford, UK; 9 Department of Chemistry, University of Oxford, Oxford, OX1 3TA UK; 10 Central Laser Facility, Research Complex at Harwell, STFC Rutherford Appleton Laboratory, UKRI, Harwell Campus, Didcot, OX11 0QX UK; 11 Serum Institute of India Pvt. Ltd., 212/2, Hadapsar, Pune, 411028 India; 12 Department of Microbiology and Infectious Diseases, Oxford University Hospitals NHS Foundation Trust, Oxford, OX3 9DU UK; 13 NIHR Oxford Biomedical Research Centre, Oxford University Hospitals NHS Foundation Trust, Oxford, OX3 9DU UK; 14 Department of Bioscience, School of Health, Sport and Bioscience, University of East London, Water Lane, London, E15 4LZ UK; 15 Agilent Technologies LDA UK, Becquerel Avenue, Didcot, OX11 0RA UK; 16 Regulation and Safety Unit, Regulation and Prequalification Department, Health Systems, Access and Data Division, World Health Organization (WHO), Geneva, Switzerland; 17 Present Address: The Francis Crick Institute, London, NW1 1AT UK; 18 Imperial College Healthcare NHS Trust, London, W2 1NY UK; 19 Imperial College London, London, SW7 2AZ UK; 20 Present Address: Exeins Health Initiative, Jakarta, 12870 Indonesia

**Keywords:** Biochemistry, Biological techniques, Biotechnology, Chemistry, Health care, Medical research

## Abstract

For many decades, there have been numerous reported cases of falsified liquid medical products, including vaccine and insulin preparations worldwide, but to date, there has been a lack of affordable and accessible analytical methods for biological medicines and vaccine authenticity testing. A conventional clinical chemistry analyser (Abbott Architect c16000) was used to determine the concentrations of analytes in genuine liquid biological products (vaccines and insulin) and falsified vaccine surrogates. Eight analytes were measured for each sample: sodium, potassium, chloride, calcium, magnesium, phosphate, glucose and protein. Each genuine liquid product had unique concentrations of analytes when tested using the eight methods applied, allowing clear differentiation from the falsified surrogates. In a blinded study, reproducibility was significantly high when the samples were run intra- and inter-batch up to 9 times over 9 different days, and it was possible to identify most of the samples by analyte presence alone. Imprecision was < 1.0 CV% for ion-selective electrode methods and typically < 5 CV% for spectrophotometric methods. A decision tree was created which was able to identify all samples. We demonstrate for the first time that a conventional clinical chemistry analyser provides a low-cost method to accurately differentiate genuine products from falsified surrogate liquid medicines and vaccines. This novel method has the potential to be used globally due to widespread use of clinical chemistry analysers in hospitals across the world, including in low- and middle-income countries where many cases of falsified medicines have been identified.

**Supplementary Information:**

The online version contains supplementary material available at 10.1038/s41598-026-37281-9.

## Introduction

One in ten medical products in low- and middle-income countries (LMICs) are estimated to be either substandard or falsified^1^. Substandard products are those which, although authorised, fail to meet either their quality standards or specifications, or both. In contrast, falsified products are those with a composition or source that is deliberately and/or fraudulently misrepresented through criminal activities^1^. Such substandard and falsified (SF) medical products pose significant, but neglected, health threats as the disease or condition will not be effectively prevented or managed, and/or the product may cause adverse health effects without a quality assurance system implemented^2^. This may lead to ineffective treatment, toxicity, and harm to patients, their communities, governments and the pharmaceutical industry. They also risk reduced public confidence, reducing trust in genuine products^3^.

A range of reference analytical techniques can be applied to assess the quality of medicines and thereby the detection of falsified and substandard products. These include optical microscopy, X-ray fluorescence, infrared spectroscopy, Raman spectroscopy, gas chromatography and mass spectrometry^4–6^. One of the challenges of applying these techniques in practice is access to the costly instrumentation and analytical expertise required for their operation in geographically remote or rural areas, especially in LMICs where the SF medicines and vaccines are more often found. There has been increased interest in accessible screening devices to detect SF vaccines and liquid medicines (such as insulin and cough medicines) in supply chains^7^ to rapidly detect them and improve selection of samples for reference pharmacopeial testing. The Vaccine Identity Evaluation Consortium has been evaluating novel techniques for detecting SF vaccines in supply chains, including spatially offset Raman spectroscopy (SORS)^8^, rapid diagnostic tests^9^, and matrix-assisted laser desorption ionization time-of-flight mass spectrometry (MALDI-ToF MS), which is available in many hospitals^10,11^. Here we introduce an inexpensive alternative technique which could be used globally for the detection of falsified vaccines and liquid medicines.

The chemical composition of vaccines and many pharmaceutical solutions includes the active vector/ingredient, and buffering and stabilising excipients, all of which, in genuine materials, are of highly purified grade and at precise concentrations. For any individual liquid biological product, it should therefore be possible to detect and measure inorganic ions, protein(s) and lipid components that are specific for a particular product. Many of these components can be detected and their concentrations measured with methods that are routinely available on clinical laboratory analysers used in hospitals and clinical laboratories worldwide. Whilst these clinical analyser methods may not individually have the analytical sensitivity or specificity of instrumentation used in the reference pharmaceutical analysis setting, we hypothesised that they may allow a multianalyte approach to detect falsified materials using an analyte concentration pattern recognition approach. Importantly, this approach would be greatly facilitated by clinical analysers being widely available and found in most hospitals worldwide, including in LMICs where there have been most cases of falsified medicines.

In this proof-of-concept study, our aim was to test whether repurposing of a clinical chemistry analyser, a low-cost technique available in many hospitals around the world, could be used to differentiate genuine vaccines and liquid medicines from falsified versions by using liquid surrogates which have previously been found or are likely to be found in falsified products. We provide evidence that this approach is accurate and has the potential to be deployed for the detection of falsified vaccines and insulin in risk-based supply chain surveillance.

## Results

**Fig. 1:**
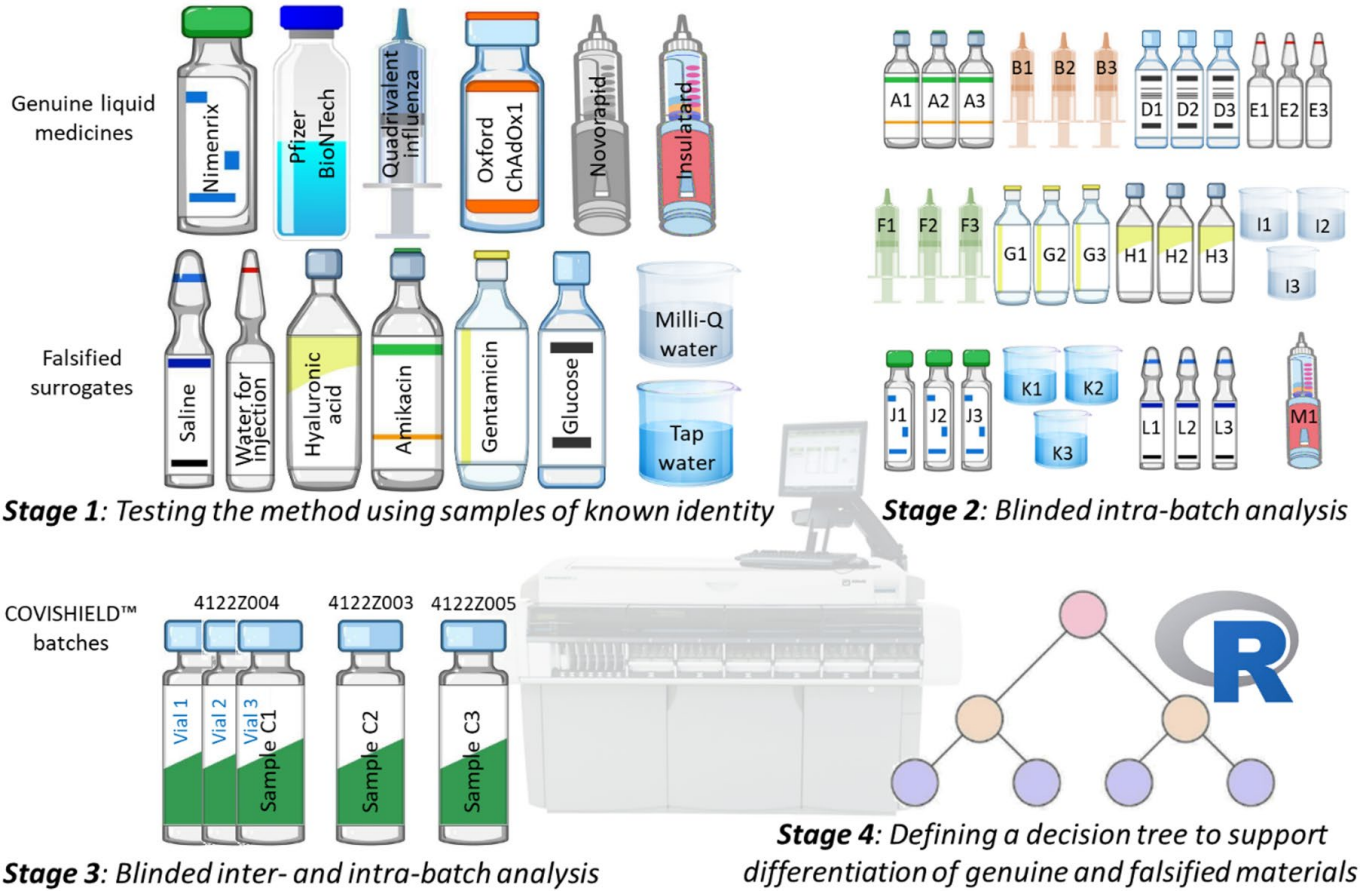
The four stages used in this study to evaluate the biochemical profiling method to differentiate genuine vaccines and insulin from falsified surrogates.

The biochemical profiling method used to differentiate genuine vaccines and insulin from falsified surrogates was evaluated in four stages (Fig. 1).

### Stage 1: testing the method using samples of known identity

Genuine vaccines, insulin and falsified vaccine surrogates were initially run on the clinical chemistry analyser to test if the method could establish a unique analyte concentration pattern for each sample. The responses of the eight methods observed in the test solutions are presented in Table S1 and the mean concentrations are shown in Table 3. Each sample had a unique panel of responses relative to the eight methods applied with respect to being, or not being, detected and quantified. When detected and quantified, the results were within the working range except for the glucose concentration of 5% glucose, which was above the upper limit of linearity. Importantly, when the response was within the working analytical concentration, good reproducibility was noted for all samples tested (Table 1). Reproducibility was lowest for the ion-selective electrode methods (sodium, potassium and chloride), with 16 of the 18 precision measurements being below 2.0 CV% across the concentration ranges in the materials tested. The spectrophotometric methods had slightly higher reproducibility with 10 of 11 precision measurements being < 5.0 CV%, with one outlier, for protein measurement of Novorapid, at 28 CV%.

**Table 1:** Biochemical methods used with their analytical characteristics and imprecision (expressed as CV%). For the instrument reporting range, the lowest value is based on the limit of quantitation and the highest value is based on the upper limit of linearity.

	Test analyte	Method Principle	Instrument reporting range	Coefficient of variation achieved across all materials tested
1	Sodium	Ion-selective electrode	20 to 400 mmol/L	0.34% at 150.9mmol/L; 0.31% at 146.9mmol/L; 0.51% at 157.0mmol/L; 0.56% at 304.7mmol/L; 1.44% at 23.6mmol/L; 2.34% at 32.5mmol/L; 1.68% at 31.9mmol/L; 0.14% at 104.1mmol/L; 0.34% at 139.2mmol/L; 4.2% at 35.3mmol/L
2	Potassium	Ion-selective electrode	1 to 300 mmol/L	0.00% at 4.2mmol/L
3	Chloride	Ion-selective electrode	20 to 300 mmol/L	0.41% at 154.0mmol/L; 0.42% at 139.9 mmol/L; 0.51% at 161.6mmol/L; 1.3% at 20.4mmol/L; 0.66% at 95.8mmol/L; 0.31% at 140.2 mmol/L; 0.63% at 40.0mmol/L
4	Calcium	Arsenazo dye	0.50 to 6.00 mmol/L	2.23% at 2.51mmol/L; 0.00% at 0.83mmol/L
5	Magnesium	Enzymatic reaction with isocitrate dehydrogenase	0.74 to 10.83 mmol/L	0.82% at 1.00mmol/L
6	Phosphate	Ammonium molybdate, UV detection	1.50 to 60.14 mmol/L	1.24% at 7.92mmol/L; 1.9% at 6.79mmol/L; 1.91% at 13.2mmol/L; 1.38% at 6.4mmol/L
7	Glucose	Enzymatic reaction using glucose hexokinase	0.06 to 44.40 mmol/L	No imprecision estimate, as all materials tested were negative with the exception of a glucose solution with a concentration above the upper concentration detectable even when applied using the standard dilution protocol used in clinical practice.
8	Protein	Denaturation using benzethonium chloride	68 to 2,000 mg/L	3.06% at 246 mg/L; 4.14% at 726 mg/L; 0.94% at 75.5 mg/L; 28.0% at 944.50 mg/L; 2.88% at 132 mg/L

**Table 3:** Analyte profiles of the test materials analysed intra-batch in stages 1 and 2 for eight test parameters using a clinical chemistry analyser. Values in bold indicate if the analytes could be detected and quantified showing their mean concentration followed by standard deviation in parentheses except for glucose which had a glucose concentration higher than the upper limit of linearity. All other values were below the limit of quantitation (LoQ), and the lower LoQ is shown as in Table 1.

Sample	Stage	Sodium mmol/L	Potassium mmol/L	Chloride mmol/L	Calcium mmol/L	Magnesium mmol/L	Phosphate mmol/L	Glucose mmol/L	Protein mg/L
Oxford COVID-19 ChAdOx1-S	1	**35.3 (0.3)**	< 1.0	**40.0 (0.3)**	< 0.50	**1.00 (0.01)**	< 1.50	< 0.06	< 68
Pfizer BioNTech COVID-19	1	**139.2 (0.5)**	< 1.0	**140.2 (0.4)**	< 0.50	< 0.74	< 1.50	< 0.06	**132 (4)**
Quadrivalent Influenza	1	**146.9 (0.5)**	**4.2 (0.0)**	**139.9 (0.4)**	< 0.50	< 0.74	**7.92 (0.10)**	< 0.06	**247 (8)**
Nimenrix	1	**157.0 (0.8)**	< 1.0	**161.6 (0.8)**	< 0.50	< 0.74	< 1.50	< 0.06	**76 (1)**
	2	**159.4 (1.3)**	< 1.0	**164.4 (1.4)**	< 0.50	< 0.74	< 1.50	< 0.06	< 68*
Pneumovax 23	2	**143.8 (0.7)**	< 1.0	**145.0 (1.0)**	< 0.50	< 0.74	< 1.50	< 0.06	**180.3 (7.4)**
Engerix B	2	**155.6 (1.5)**	< 1.0	**143.2 (0.9)**	< 0.50	< 0.74	**8.47 (0.6)**	< 0.06	< 68*
Novorapid	1	**32.5 (0.8)**	< 1.0	**20.4 (0.3)**	< 0.50	< 0.74	**6.79 (0.1)**	< 0.06	**945 (264)**
Insulatard	1	**31.8 (0.5)**	< 1.0	< 20.0	< 0.50	< 0.74	**13.2 (0.3)**	< 0.06	< 68
	2	**28.1 (0.3)**	< 1.0	< 20.0	< 0.50	< 0.74	**13.0 (0.2)**	< 0.06	< 68
Milli-Q water	1	< 20.0	< 1.0	< 20.0	< 0.50	< 0.74	< 1.50	< 0.06	< 68
	2	< 20.0	< 1.0	< 20.0	< 0.50	< 0.74	< 1.50	< 0.06	< 68
Tap water	1	< 20.0	< 1.0	< 20.0	**2.51 (0.06)**	< 0.74	< 1.50	< 0.06	< 68
	2	< 20.0	< 1.0	< 20.0	**2.07 (0.02)**	< 0.74	< 1.50	< 0.06	< 68
Water for injection	1	< 20.0	< 1.0	< 20.0	< 0.50	< 0.74	< 1.50	< 0.06	< 68
	2	< 20.0	< 1.0	< 20.0	< 0.50	< 0.74	< 1.50	< 0.06	< 68
Saline	1	**150.9 (0.5)**	< 1.0	**154.0 (0.4)**	< 0.50	< 0.74	< 1.50	< 0.06	< 68
	2	**152.3 (0.9)**	< 1.0	**155.2 (1.1)**	< 0.50	< 0.74	< 1.50	< 0.06	< 68
Glucose	1	< 20.0	< 1.0	< 20.0	< 0.50	< 0.74	< 1.50	**> 222.00**	< 68
	2	< 20.0	< 1.0	< 20.0	< 0.50	< 0.74	< 1.50	**> 222.00**	< 68
Hyaluronic acid	1	< 20.0	< 1.0	< 20.0	< 0.50	< 0.74	< 1.50	< 0.06	**726 (30)**
	2	< 20.0	< 1.0	< 20.0	< 0.50	< 0.74	< 1.50	< 0.06	**752 (61)**
Amikacin (Tillomed)	1	**304.7 (1.7)**	< 1.0	< 20.0	< 0.50	< 0.74	< 1.50	< 0.06	< 68
Amikacin (Hospira)	2	**276.9 (4.4)**	< 1.0	< 20.0	**0.67 (0.02)**	< 0.74	< 1.50	< 0.06	< 68
Gentamicin	1	**23.6 (0.3)**	< 1.0	< 20.0	**0.83 (0.00)**	< 0.74	< 1.50	< 0.06	< 68
	2	**20.2 (0.24)**	< 1.0	< 20.0	**1.07 (1.01)**	< 0.74	< 1.50	< 0.06	< 68

* Protein concentration < LoQ (< 68 mg/L) for most, but not all, of the runs.

### Stage 2: blinded study

Genuine vaccines, insulin and falsified vaccine surrogates were provided to the operator of the clinical chemistry analyser in glass vials labelled with a code but without the identity of the samples (Fig. 1). The responses of the eight methods observed for these blinded samples are presented in Table S2 and the mean values are shown in Table 3. The additional vaccines, Engerix B and Pneumovax 23, had unique panels of responses in a similar way to the other vaccines. Samples already analysed in Stage 1 gave similar responses. For most of the initial Engerix B runs, no protein was detected. However, protein was detected with 333 mg/L for the last run of the sample in syringe 1 and 288, 356, 152 mg/L for the last three runs of syringe 2 (Table S2). As in Stage 1, good reproducibility was noted for all materials tested. One-way ANOVA indicated good consistency (*p* > 0.05) in analyte concentrations between the three different vials, syringes and ampoules for all runs except for amikacin (*p* < 0.001) where vial 2 showed slightly lower concentrations for sodium and calcium, only 3 and 6% lower, respectively, compared to vials 1 and 3. The analyte concentrations between vials 1 and 3 were very similar (*p* > 0.05).

### Stage 3: analysis of different vaccine batches

Three different batches of COVISHIELD™ vaccine were analysed with the operators blinded as in Stage 2. The responses of the eight methods, along with *p*-values for one-way ANOVA, are presented in Table S3 and the mean values are shown in Table 4. Similar concentrations were observed for all eight analytes when analysing the vaccine in three different vials of the same batch (4122Z004). All analytes showed good consistency between the three batches (*p* > 0.05) with the exception of protein (*p* < 0.001), which was below the limit of quantitation in batch 4122Z005, 79.1 mg/L for batch 4122Z003 and 96.1 mg/L for batch 4122Z004.

**Table 4:** Inter-batch data for the COVISHIELD™ vaccine in the stage 3 analysis. Analyte profiles are shown for the eight test parameters. Values in bold indicate if the analytes could be detected and quantified showing their mean concentration followed by standard deviation in parentheses. All other values were below the limit of quantitation (LoQ), and the lower LoQ is shown as in table 1. The intra-batch data for COVISHIELD™ is shown in table S3.

*Batch*	Sodium mmol/L	Potassium mmol/L	Chloride mmol/L	Calcium mmol/L	Magnesium mmol/L	Phosphate mmol/L	Glucose mmol/L	Protein mg/L
4122Z004	**34.5 (0.3)**	< 1.0	**39 (0.5)**	< 0.50	**1.02 (0.03)**	< 1.50	< 0.06	**96.1 (5)**
4122Z003	**34.6 (0.4)**	< 1.0	**39.1 (0.5)**	< 0.50	**1.04 (0.03)**	< 1.50	< 0.06	**79.2 (5)**
4122Z005	**34.6 (0.3)**	< 1.0	**39.1 (0.5)**	< 0.50	**1.02 (0.03)**	< 1.50	< 0.06	< 68

### Stage 4: A decision tree for identification of genuine and falsified products

**Fig. 2:**
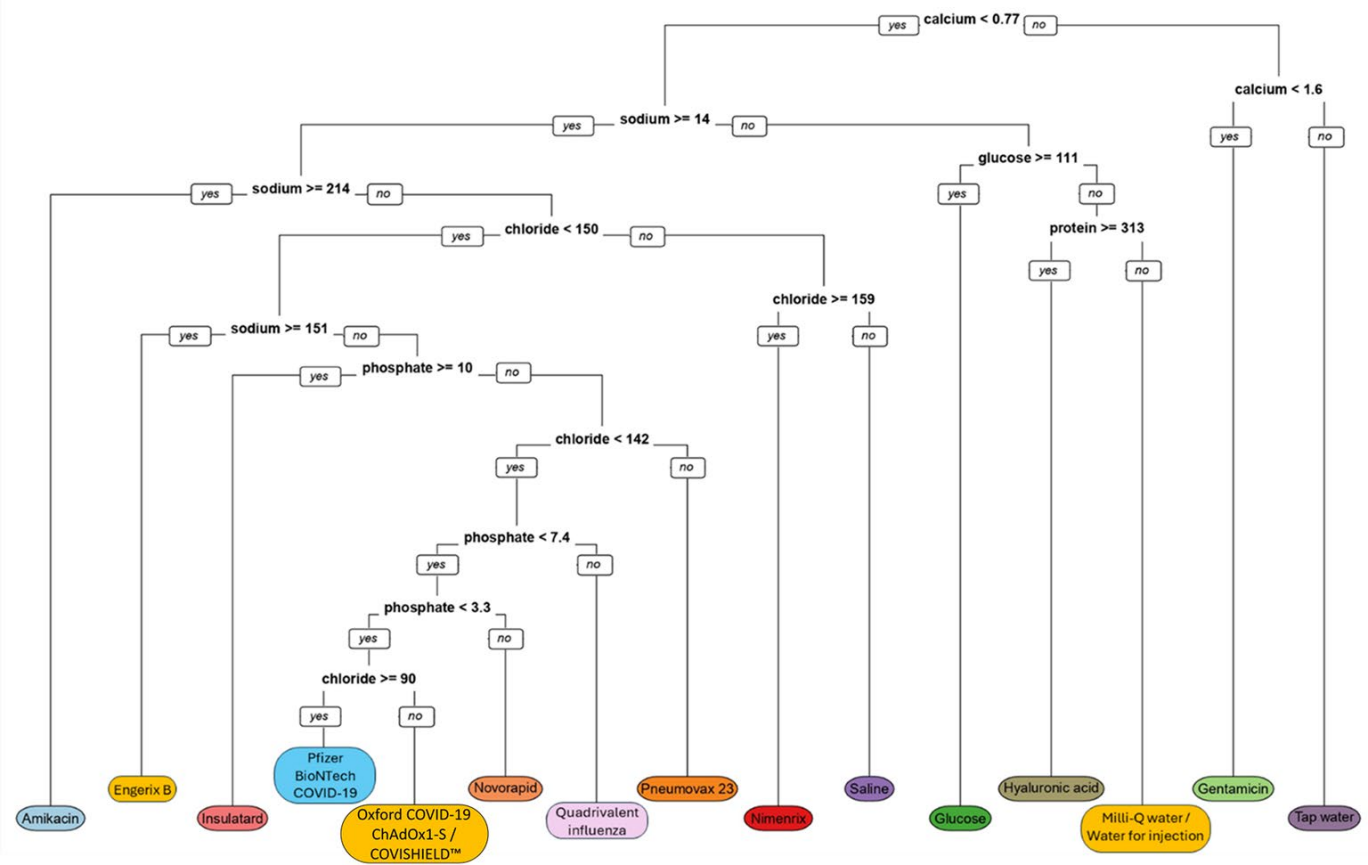
Decision tree for identification of an unknown test material to determine identification.

Results were analysed using recursive partitioning to build a classification model and construct a decision tree^12,13^. The tree provides a proof-of-concept visual representation of how the different biochemical signatures may be interpreted in a simple binary algorithm to identify the different vaccines (Fig. 2).

## Discussion

There is a global and urgent need for accessible screening devices to detect SF liquid medicines in supply chains, with mutliple alerts for falsified vaccines and insulin (Table 2). The composition, concentration and quality of excipients in genuine injectable vaccines and liquid medicines, such as insulin, are tightly controlled in the manufacturing process, which would be much less likely in falsified products. Liquid medicines often have constituents that have physiological relevance and are routinely analysed in blood and/or urine to support clinical patient management. In this study, we have used a clinical chemistry analyser, available in hospital settings worldwide, to provide a profile using simple biochemical methods. While the method cannot confirm or differentiate the components present in each injectable medicine, it can accurately measure the concentrations of eight analytes which serves as a distinctive profile to confirm authenticity. The profile response across eight simple methods with respect to authentic vaccines and insulin differs from those of solutions used in falsified medical products. The responses were reproducible on the same instrument over time (Table 3, S1 and S2). For the ion-selective electrode methods, the imprecision was typically < 2.0 CV%, which was consistent with the imprecision commonly seen when testing clinical specimens. The typical reagent and consumable costs are <£10 for the eight-test profile undertaken here. The concentrations of analytes between different syringes and vials intra-batch were found to be very consistent (Table S2). We do not expect this technique to be accurate in the detection of substandard vaccines and liquid medicines, due to errors in manufacturing, unless gross, in supply chains.

**Table 2:** The genuine vaccines, insulin and falsified vaccine surrogates tested in this study.

Sample type	Sample	Manufacturer/Source	Composition	Rationale for inclusion	Target pathogen/disease
Vaccine	Oxford COVID-19 ChAdOx1-S vaccine^a^	The Clinical BioManufacturing Facility, University of Oxford	histidine, magnesium chloride, polysorbate 80, ethanol, sucrose, sodium chloride, edetate disodium, recombinant replication-defective chimpanzee adenovirus expressing the SARS-CoV-2 S surface glycoprotein	Falsified Oxford-AstraZeneca COVID-19 vaccine has been identified in Iran^19^	COVID-19
	Pfizer BioNTech COVID-19 vaccine^a^	Pfizer BioNTech	4-hydroxybutyl)azanediyl)bis(hexane-6,1-diyl)bis(2-hexyldecanoate) (ALC-0315), 2[(polyethylene glycol)-2000]-N, N-ditetradecylacetamide (ALC-0159), 1,2-distearoyl-sn-glycero-3-phosphocholine, cholesterol, sucrose, potassium chloride, monobasic potassium phosphate, sodium chloride, dibasic sodium phosphate dehydrate, tozinameran mRNA	Falsified Pfizer BioNTech COVID-19 vaccine has been identified in Iran^20^	
	COVISHIELD™ COVID-19 vaccine^c^	Serum Institute of India	L-histidine, L-histidine hydrochloride monohydrate, magnesium chloride hexahydrate, polysorbate 80, ethanol, sucrose, sodium chloride, disodium edetate dihydrate, recombinant, replication-deficient chimpanzee adenovirus vector encoding the SARS-CoV-2 Spike (S) glycoprotein	Falsified COVISHIELD™ vaccine has been identified in India, Myanmar and Uganda^21^	
	Quadrivalent Influenza^a^	Sanofi	sodium chloride, potassium chloride, disodium phosphate dihydrate, potassium dihydrogen phosphate, influenza virus (inactivated, split)	Influenza vaccines made by Sanofi have been falsified in Brazil^22^	Influenza
	Nimenrix^a, b^	Pfizer	sucrose, trometamol, sodium chloride, *Neisseria meningitidis* polysaccharides	Other vaccines for bacterial meningitis, such as Mencevax, have been falsified^23^	Meningococcal disease
	Pneumovax 23^b^	Merck Sharp & Dohme	phenol, sodium chloride, pneumococcal polysaccharide serotypes		Pneumococcal pneumonia/ meningitis
	Engerix B^b^	GlaxoSmithKline	sodium chloride, disodium phosphate dihydrate, sodium dihydrogen phosphate, aluminium hydroxide, hepatitis B surface antigen	Falsified hepatitis B vaccine has been identified in Uganda^24^	Hepatitis B
Insulin	Novorapid^a^	Novo Nordisk	insulin aspart, glycerol, phenol, metacresol, zinc chloride, disodium phosphate dihydrate, sodium chloride, hydrochloric acid, sodium hydroxide, insulin aspart	Falsified versions of other brands of insulin (e.g. Knowit Insulin in Nigeria) have been identified^25^	Diabetes
	Insulatard^a, b^	Novo Nordisk	insulin, zinc chloride, glycerol, metacresol, phenol, disodium phosphate dihydrate, sodium hydroxide, hydrochloric acid, protamine sulfate, human isophane insulin		
Falsified vaccine surrogates	Milli-Q water^a, b^	Milli-Q system made by Merck Millipore	Ultrapure water	From a Milli-Q Direct 8 water purification system	N/A
	Tap water^a, b^	Department of Biochemistry, Oxford	Tap water from biochemistry laboratory	Water from Thames Water mains water supply	
	Water for injection^a, b^	Demo S.A Pharmaceutical Industry	Sterile water for preparation of a medicine intended for injection or infusion	Water for injection in plastic ampoules	
	Saline^a, b^	Demo S.A Pharmaceutical Industry	0.9% w/v sodium chloride in water for injection	Surrogate for falsified COVID-19 vaccines intercepted in China and India (Mumbai)^26^	
	Glucose^a, b^	B/Braun	D-glucose 5.0% w/v	Falsified COVID-19 vaccine in Binondo, Philippines was dextrose^26^	
	Hyaluronic acid^a, b^	Guangzhou Ailian Cosmetic Co Ltd.	Anti-wrinkle serum containing water, glycerine, propylene glycol, methylisothiazolinone, bis(hydroxmethyl) imidazolidinyl urea, iodopropynyl butylcarbamate, disodium EDTA, xanthan gum, sodium hyaluronate	Surrogate for falsified COVID-19 vaccines intercepted in Poland. The precise formulation and form of intercepted hyaluronic product unknown apart from it being reported being an anti-wrinkle formulation^26^	
	Amikacin − 250 mg/mL^a^	Tillomed Laboratories Ltd.	amikacin sulphate, sodium metabisulfite, sodium citrate dihydrate, sulfuric acid and water for injection	Surrogate for falsified COVID-19 vaccines intercepted in India^26^	
	Amikacin − 250 mg/mL^b^	Hospira	amikacin sulphate, sodium citrate, sodium metabisulphite and water for injection		
	Gentamicin - 40 mg/mL^a, b^	Demo S.A.	gentamicin sulphate, sodium metabisulfite, disodium edetate	Surrogate for falsified non-COVID vaccines intercepted in Indonesia^26^	

^a^ Samples used in Stage 1, ^b^ Samples used in Stage 2, ^c^ Samples used in Stage 3.

The specific analyser on which the tests were determined was an Abbott analyser, and this has enabled demonstration of the proof-of concept of this approach. Equivalent testing for all the methods mentioned are available from a range of manufacturers, which could be utilised in the same manner, and all provide high throughput (hundreds of tests per hour) and use low specimen volumes (typically around 100 µL). It is also of note that some vaccines, such as mRNA vaccines and those with lipid-based adjuvants, contain lipid components which could also be determined. Chemistry analysers can also be set up for methods beyond those provided by the primary instrument provider for the range of analytes described. If a spectrophotometric method can be developed, it could, in principle, be applied to these systems as user-defined methods^14^ to provide unique profiles for detection of a much wider range of SF products to meet evolving trends in falsification^15^.

All methods used, with the exception of protein analysis (where a specific urine method was applied), can be utilised in two modes, one optimised for analysing serum specimens and the other optimised for urine specimens. The method differences may utilise different sample volumes, different calibration strategies, and consequently different working ranges applicable to clinical samples. As the analyte matrix of vaccines and medication solutions are generally simple aqueous solutions with low concentrations of protein relative to those in serum, we used the testing mode designed for urine. For most tests this provides a wider working range of concentrations and can be used with matrix-matched calibrators for improved analytical accuracy, for example the methods used in the current study for sodium, potassium and chloride. In general, this extends the upper limit of linearity at the expense of increasing the lower limit of quantitation. This achieved good differentiation of the products profiles, but if the profiling of any suspected material required greater sensitivity, i.e. for detection at lower concentrations, the serum mode of analysis could be utilised.

All the methods utilised were electrochemical or spectrophotometric methods, available on semi-automated or automated chemistry analysers which are on the WHO model list of essential in-vitro diagnostics^16^. Therefore, testing capability is widespread worldwide including in LMICs where many cases of falsified medicines and vaccines have been identified. Whilst not investigated in this study, many of these experiments could be undertaken using handheld point-of-care devices. This is an additional analytical option that could be explored for application in the field.

For the detection of vaccine excipients, we have recently demonstrated that SORS can be used to generate spectra of sucrose and ethanol^8^ while MALDI-ToF is suitable for detecting polysorbate 80 and histidine^11^. While the clinical chemistry analyser cannot detect these excipients, it was able to successfully detect and quantify up to eight different analytes at low cost, which serves as a signature for vaccines and insulin products. We have also shown that low-cost rapid diagnostic tests can detect the active ingredients in vaccines^9^. While the clinical chemistry analyser could not specifically detect active ingredients in vaccines, it was able to confirm the presence of proteins when present, e.g. successful detection of HBsAg in Engerix B (Table S2). There will be no single device to help screen medicines and vaccines throughout the supply chain and different devices would be required at various stages and locations, also ensuring comprehensive monitoring. In detecting the criminal activity of falsification, diverse technologies will be needed, as in checking for dangerous agents in airline security, to inhibit sophisticated criminals countering these systems. Clinical chemistry analysers, SORS and MALDI-ToF MS could help to screen liquid medicines in proximal locations of the supply chain whereas SORS and rapid diagnostic tests could help in provincial and distal locations, thereby enabling end-to-end vaccine supply chain monitoring. This highlights the complementarity of the clinical chemistry analyser and these other three approaches.

A decision tree was created which could be used to successfully determine not only the identity of the genuine liquid product but also could help to determine the falsified sample. By analysing which analytes were observed for each sample, it was possible to identify most of them even without prior knowledge of the excipient concentrations. The mean concentrations for the blinded samples in Stage 2 was used to correctly identify all of the samples with information only on the excipients and not their concentrations, except for Nimenrix and Pneumovax 23 which had the same analytes present. However, these vaccines could be successfully differentiated by taking into consideration the analyte concentrations determined for Nimenrix in the Stage 1 analysis. The application of a decision tree in this study allowed a simple visual flowchart for the identification of the different samples. However, further development would be required to implement the analysis in a real-world setting, due to the risks of false negatives/positives from samples outside of the sample set included in this study. Equally, alternative predictive modelling tools would need to be explored with larger datasets and additional types of vaccines and vaccine surrogates. For example, a chemometric approach such as Principal Component Regression (PCR) and Partial Least Squares Regression (PLS) can help to automate sample classification in developed countries with well-equipped clinical settings. In LMIC settings, a simple “cue card” verification-focused approach could be used where a cue card/checklist acts as a reference with the expected analyte concentrations for each known biologic/vaccine, and further evaluation undertaken if it did not demonstrate expected results.

All surrogates of falsified liquid medicines were successfully identified from the blinded data in Stage 2. As expected, the ultrapure water samples (Milli-Q water and water for injection), had no analytes present. Tap water did not have any of the measured analytes present except for calcium which was expected since it is known to be the mineral at the highest concentration^17^. The water dispensed from the tap in Stages 1 and 2 were collected on different days and the analyser was able to detect minor differences between these different dispenses (2.51 mmol/L calcium in Stage 1 and 2.07 mmol/L in Stage 2). The 5% glucose sample was the only sample which had glucose and at a high level above the upper limit of linearity (> 222 mmol/L). This was expected since the 5% glucose sample is expected to be 278 mmol/L. Saline is sodium chloride in water and therefore was easily identified since there was only one sample where sodium and chloride ions were present, at the same concentration and was close to the expected concentration of 154 mmol/L for both ions. The antibiotics, amikacin and gentamicin, contain sodium metabisulfite and sodium citrate dihydrate and were the only samples which contained sodium ions but lacked other ions such as chloride, potassium and phosphate. Amikacin from two different manufacturers were tested and while both showed the presence of sodium ions, only one additionally showed a very low concentration of calcium in the same way as gentamicin.

Insulin samples were also tested in Stages 1 and 2. Insulatard contains disodium phosphate dihydrate and could be easily identified since it was the only sample with sodium and phosphate and no other ions. In both Stage 1 and 2, no protein was identified for Insulatard, even though it is known to contain a high concentration of insulin (3.5 mg/mL). However, 945 mg/L of protein was detected for Novorapid, another insulin product also known to contain 3.5 mg/mL of insulin. Differences in the actual amount of protein in the sample and measured values are expected since the analyser method has been optimised to be used for urine samples and not insulin or vaccines where the sample matrix is considerably different and could interfere with the assay. These differences are not a problem since the pattern of analyte concentrations are reproducible and could be used to confirm authenticity. The imprecision measurements when analysing Novorapid, being 28 CV%, were atypical of performance in routine clinical practice when testing human urine and this may suggest that characteristics of the sample may contribute to variability and therefore requires further investigation.

For the Stage 2 analysis, the Oxford COVID-19 ChAdOx1-S could be identified simply by analyte presence without analysing their concentrations. This vaccine is known to contain magnesium ions (magnesium chloride) and as expected was the only sample in Stage 2 where magnesium was identified. Sodium and chloride ions were also identified in this sample which was expected since the vaccine also contained sodium chloride and edetate disodium. Pfizer BioNTech COVID-19 vaccine contains potassium chloride, monobasic potassium phosphate, sodium chloride and dibasic sodium phosphate dihydrate. The sodium and chloride ions were detected but not potassium or phosphate ions due to low concentrations below the analyser’s lower limit of quantitation. This vaccine is known to not contain any protein, but the analyser estimated the protein concentration in the vaccine to be 132 mg/L. The analyser’s method for protein uses benzethonium chloride to denature proteins which are then detected by turbidimetry. However, benzethonium chloride is a cationic surfactant which may have interacted with the lipids and disrupted the lipid nanoparticles in the mRNA vaccine. This false positive for protein is therefore expected and would not be a problem with the analyser’s intended and optimised use of analysing urine samples. Also, this false positive is not a problem for confirming the authenticity of this mRNA vaccine since the pattern of analyte concentrations, including the false positive for protein, was reproducible. Alternative urine protein methods are dye based, rather than turbidimetric, and may offer additional flexibility within a test profile to detect falsified vaccines. These alternative protein methods may provide greater reproducibility within the context of the current application.

Some of the blinded non-COVID-19 vaccine samples in Stage 2 were identified simply by noting which analytes were present without the need to analyse the analyte concentrations. Quadrivalent influenza vaccine contains inactivated influenza virus, sodium chloride, potassium chloride, disodium phosphate dihydrate and potassium dihydrogen phosphate. As expected, protein, sodium, potassium, chloride and phosphate were identified and, since this was the only sample in which potassium was detected, it could be easily identified among both other vaccines and falsified surrogates. Engerix B contains sodium chloride, disodium phosphate dihydrate and sodium dihydrogen phosphate. As expected, the ions observed were sodium, chloride and phosphate which were only observed for this sample making it easy to identify among the other blinded samples. Engerix B contains 20 µg/mL hepatitis B surface antigen (HBsAg). This concentration of HBsAg is below the analyser’s lower limit of quantitation for protein (68 µg/mL) and therefore as expected no protein was observed for most of the runs. However, protein was detected in the last 1–3 runs for the vaccines from two syringes with high, and variable, concentrations ranging from 152 to 356 µg/mL (Table S2). HBsAg is adsorbed onto aluminium hydroxide which settles to the bottom of the syringe by gravity if the syringe is left undisturbed. This explains why protein was only detected in the last few runs since the HBsAg adsorbed onto aluminium hydroxide would have settled to the bottom of the tube in the analyser resulting in concentrations above the 20 µg/mL for HBsAg.

Nimenrix and Pneumovax 23 are both sodium chloride-containing vaccines, and the presence of sodium, chloride and protein were detected in both. Although these vaccines had the same analytes, they could be easily differentiated since Nimenrix is lyophilised and requires reconstitution in saline whereas Pneumovax 23 is a ready to use liquid vaccine. However, if two liquid vaccines had the same ions in this way, it would be difficult to confirm their identity unless there was prior knowledge of the concentrations of these analytes. By referencing the analyte concentrations determined for Nimenrix in the Stage 1 study, it was possible to correctly identify this vaccine in the Stage 2 blinded study (Table 3). Liquid vaccines are made up precisely by the vaccine manufacturer but in the case of dried vaccines, such as Nimenrix, there is the possibility of minor variation since the final concentration of analytes may vary to a small degree if a slightly different volume of saline is added by the end user when reconstituting. However, three different vials of Nimenrix were analysed and each vial was made up separately with the provided saline syringe. Despite being made up separately with saline, the concentrations of both sodium and chloride were very consistent among vials (*p* > 0.05; see Table S2). Although the average difference in sodium and chloride ions between these vaccines was only 15.6 and 19.4 mmol/L, respectively, an independent two-sample t-test comparing Nimenrix and Pneumovax showed *p* < 0.05 for both sodium (*p* = 2.07 × 10⁻¹⁷) and chloride (*p* = 1.86 × 10⁻¹⁸) ions confirming that there were significantly different. Analysis of sodium and chloride ions in different batches of Nimenrix and other powdered vaccines requires further investigation.

In the Stage 3 analysis the method was tested on three different vials of the same batch (intra-batch) and three different batches (inter-batch) of the COVISHIELD™ vaccine (Table 4 and Table S3). The intra-batch analysis showed similar concentration for all eight analytes. The panel of responses for COVISHIELD™ were similar to the Oxford COVID-19 ChAdOx1-S vaccine (i.e. with magnesium, sodium and chloride) and overlapped in the decision tree (Fig. 2) which was expected since both are ChAdOx1 vaccines with a similar excipient composition (Table 2). The inter-batch analysis showed good consistency for the concentration of sodium, chloride and magnesium. This confirms excellent batch-to-batch consistency which is expected since vaccine manufacturers tightly control the concentrations of excipients among batches. We also analysed these three batches in previous studies and found them to have very similar spectra by both SORS^8^ and MALDI-ToF MS^11^, further supporting the high level of consistency of these batches. Although excipient levels are always made up precisely, the number of viral particles in viral vector vaccines can vary. For example, the Oxford-AstraZeneca COVID-19 ChAdOx1 vaccine must have equal to or more than 0.7 × 10^11^ viral particles per mL.^18^ Therefore, the protein levels can differ among different batches, and as expected, we observed different levels of protein in the three different batches of COVISHIELD™ (Table 4; *p* < 0.001). This inter-batch data highlights that while a clinical chemistry analyser can be used to establish a library of analyte concentrations for each vaccine, care should be taken if proteins are present and their concentrations are known to vary slightly among batches. Although it was possible to identify most of the solutions in this study without prior knowledge of the excipient concentrations, we had only analysed a limited number of vaccines, insulin and falsified surrogates. Nimenrix and Pneumovax 23 are examples of vaccines with similar excipients but not at similar concentrations. There are other liquid medicines with similar excipients but at different concentrations highlighting the importance of establishing the exact concentrations of the analytes for each genuine liquid medicine.

## Conclusion

In this study, it was demonstrated that a clinical chemistry analyser, conventionally used in hospitals worldwide, could successfully determine unique analyte concentrations of authentic liquid medical products (vaccines and insulin) and distinguish these from falsified medicine surrogates. Hence, a low-cost assay using a clinical chemistry analyser could be used to detect falsified vaccines and liquid medicines in supply chain surveillance. Although a decision tree was able to successfully identify all the different samples, alternative chemometric approaches need to be investigated especially with larger datasets.

## Methods

### Instrumentation and method principles

We tested a wide range of genuine vaccines, insulin and surrogate liquids found in falsified vaccines. The clinical laboratory analyser utilised was a commonly available device, the Abbott Architect c16000 analyser (Abbott Laboratories, Maidenhead, UK). Method principles were either spectrophotometric or ion-selective electrodes, developed and optimised by the manufacturer to be used for clinical purposes and had analytical concentration ranges designed to meet the clinical requirements of testing biological specimens. The method optimised for urine specimens was used. Specific performance characteristics for these methods are defined according to standard protocols recommended by the Clinical and Laboratory Standards Institute (CLSI) (prior to 2005 known as the National Committee for Clinical Laboratory Standards (NCCLS)).

Selected analytical characteristics of the eight methods applied in the current study, determined using CLSI protocol NCCLS EP6-P are presented in Table 1. For both spectrophotometric and ion selective electrode methods the lower and upper limits of linearity are defined.

### Study approach

Four stages were undertaken to evaluate the detection methodology (Fig. 1). Stage one tested the method by characterising the response of the selected methods for a range of genuine vaccines and materials historically found to be used in falsified vaccines (Table 2). Each material was tested with 4 repeats, except for the Pfizer BioNTech COVID-19 vaccine and Nimenrix for which only 2–3 replicates were possible due to limited sample availability. All samples analysed were liquids except for Nimenrix which is supplied as a dried powder. Nimenrix was reconstituted by adding the entire contents of the supplied pre-filled syringe of solvent (0.5 mL saline) to the vial containing the powder. This mixture was shaken well until the powder was completely dissolved in the solvent. Testing of all samples was conducted over the course of one week to evaluate the method and assess its reproducibility. The mean concentration of each response within the working analytical range of the test was calculated together with the standard deviation (SD). From these a percentage coefficient of variation was calculated (SD/mean*100).

The second stage involved a blinded analysis of the same falsified vaccine surrogates, Insulatard, Nimenrix and two additional vaccines (Engerix B and Pneumovax 23) which were not analysed in Stage 1. These samples were provided to the operator of the clinical chemistry analyser in glass vials labelled with a code but without the identity of the samples (Fig. 1). All samples were blinded for comparison against the responses determined in stage 1. This was a more in-depth study aimed at evaluating vial-to-vial and syringe-to-syringe reproducibility within a batch, using a greater number of replicates. Samples were analysed from three different vials/ampoules/syringes from the same batch of each vaccine and falsified surrogate, except for Insulatard, for which only a single syringe was available. Each of the three vials/syringes were analysed nine times over nine separate days, i.e. a total of 27 runs for each sample, except for Nimenrix and Pneumovax 23 where there was only enough sample for 11 and 14 repeats, respectively. The tap water used in Stage 2 consisted of three fresh dispenses, distinct from the one used in Stage 1. One-way ANOVA was carried out for all analyte concentrations above the limit of quantitation, with *p* > 0.05 indicating intra-batch consistency and no statistically significant differences.

The third stage involved analysing different batches of a vaccine in a blinded study. For this stage, the COVISHIELD™ vaccine kindly provided by the Serum Institute of India, Pvt. Ltd. was evaluated. In a similar way to Stage 2, vaccines from three different vials from the same batch (4122Z004) were analysed. In addition, one vial from two other batches (4122Z003 and 4122Z005) were analysed (Fig. 1). One-way ANOVA was carried out for all analyte concentrations above the limit of quantitation and *p* > 0.05 was used to confirm consistency intra- and inter-batch with no statistically significant difference.

Stage four focused on developing a decision tree for application in practice. R version 4.5.0 was used with the package ‘‘rpart’ to perform recursive partitioning to construct the decision tree^12^.

## Supplementary Information

Below is the link to the electronic supplementary material.

Supplementary Material 1

Supplementary Material 2

Supplementary Material 3

## Data Availability

All data generated or analysed during this study are included in this published article and its supplementary information files.
